# Olive Volatiles from Portuguese Cultivars Cobrançosa, Madural and Verdeal Transmontana: Role in Oviposition Preference of *Bactrocera oleae* (Rossi) (Diptera: Tephritidae)

**DOI:** 10.1371/journal.pone.0125070

**Published:** 2015-05-18

**Authors:** Ricardo Malheiro, Susana Casal, Sara C. Cunha, Paula Baptista, José Alberto Pereira

**Affiliations:** 1 Mountain Research Centre (CIMO), School of Agriculture, Polytechnic Institute of Bragança, Campus de Santa Apolónia, Apartado 1172, 5301–855, Bragança, Portugal; 2 LAQV@REQUIMTE/Laboratory of Bromatology and Hydrology, Faculty of Pharmacy, Porto University, Rua de Jorge Viterbo Ferreira, 228, 4050–313, Porto, Portugal; Agricultural University of Athens, GREECE

## Abstract

The olive fly, *Bactrocera oleae* (Rossi), a serious threat to the olive crop worldwide, displays ovipositon preference for some olive cultivars but the causes are still unclear. In the present work, three Portuguese olive cultivars with different susceptibilities to olive fly (Cobrançosa, Madural, and Verdeal Transmontana) were studied, aiming to determine if the olive volatiles are implicated in this interaction. Olive volatiles were assessed by SPME-GC-MS in the three cultivars during maturation process to observe possible correlations with olive fly infestation levels. Overall, 34 volatiles were identified in the olives, from 7 chemical classes (alcohols, aldehydes, aromatic hydrocarbons, esters, ketones, sesquiterpenes, and terpenes). Generally, total volatile amounts decrease during maturation but toluene, the main compound, increased in all cultivars, particularly in those with higher susceptibility to olive fly. Sesquiterpenes also raised, mainly α-copaene. Toluene and α-copaene, recognized oviposition promoters to olive fly, were correlated with the infestation level of cvs. Madural and Verdeal Trasnmontana (intermediate and highly susceptible cultivars respectively), while no correlations were established with cv. Cobrançosa (less susceptible). No volatiles with inverse correlation were observed. Volatile composition of olives may be a decisive factor in the olive fly choice to oviposit and this could be the basis for the development of new control strategies for this pest.

## Introduction

*Olea europaea* L. has registered a considerable growth and dissemination worldwide in the last decades, attracting the attention of new producing countries worldwide. Therefore productive records are being registered since the beginning of this decade, exceeding for the first time the 20 million tons barrier (20.4 million tons of olives in 2011; 20.3 million tons in 2013; [1]). Escorting such dissemination, the dispersion of *Bactrocera oleae* (Rossi) (Diptera: Tephritidae), the olive fly, is also verified, being a key pest of olives worldwide, with special importance in the Mediterranean region [2]. This dipteran causes severe olive production losses due to fruit drop [3], leads to the production of low quality olive oils [4], and olives infected by this pest cannot be used for table olives processing. Olive fly is also a vehicle of phytopathogenic agents [5, 6] leading to the appearance and development of other olives diseases. Altogether, pests and diseases are believed to reduce olives production by 15% on average [7], which means that about 3.6 million tons of olives were wasted in 2013, with olive fly being responsible for tremendous share in this loss.

Regarding olive fly infestation, olive cultivars display different susceptibilities to this pest, with some cultivars having systematically low infestation levels, while others, within the same agro-ecosystem, are usually more heavily affected [5, 8–10]. In this particular olive fly/olive tree interaction several factors, including physical, chemical and molecular aspects influence preference of olive fly towards specific olive cultivars. Concerning chemical cues, volatiles emission may exert a strong influence in olive fly varietal preference. Several works highlight the attractant and/or repellent activity of chemical volatiles in olive fly: pheromones and other semiochemicals [11, 12]; host volatiles [13, 14]; and bacterial filtrate volatiles [15]. More recently, a possible link with sesquiterpenes was also raised [16]). However, studies reflecting the susceptibility of different olive cultivars and their volatile emissions are scarce. These studies, involving cultivars with different vulnerability degrees to olive fly oviposition, could give important information about host selection causes and possible volatiles involved in the binomial *O*. *europaea*—*B*. *oleae*.

In this sense, three Portuguese olive cultivars, Cobrançosa, Madural and Verdeal Transmontana, were selected based on their susceptibility to olive fly. Olives from cvs. Madural and Verdeal Transmontana are highly susceptible to olive fly, while cv. Cobrançosa displays lower susceptibility than the others [9]. The main objective of this work was to characterize the volatile fraction of the olives during ripening by HS-SPME-GC-MS (headspace solid-phase microextraction/gas chromatography with mass spectrometry detector), while trying to establish possible relations between infestation levels observed on each cultivar with the volatiles of the drupes.

The determination of volatile composition of olives from cultivars with different susceptibility degrees to olive fly could give answers to the binomial olive fly-olive tree. By monitoring the volatile fraction and infestation levels of the different cultivars through the maturation process, important information could be retained regarding the possible existence of volatiles responsible for the olive fly attraction and/or repellence. From the results obtained, new hypothesis may be elaborated in order to turn the control of olive fly more eco-friendly and sustainable by the application of volatiles naturally present in olives.

To the authors knowledge, this is the first investigation of this kind with these cultivars, and the first study reporting possible relations between olive fly varietal preference and olive fruit volatile composition.

## Material and Methods

### Sampling

For the present study olives from three Portuguese olive cultivars, the most representative from Trás-os-Montes region (Northeast of Portugal) were assessed: *cvs*. Cobrançosa, Madural and Verdeal Transmontana. The work occurred in 2011, and samples were collected in a private organic olive grove located in Paradela (Mirandela—41°32’35.72”N; 7°07’27.17”W) (permission to carry out samples collection was kindly granted by the olive grove owner). Five trees were marked per cultivar and branches with olives were collected at six different dates: 18^th^ July; 18^th^ August; 20^th^ September; 4^th^ October; 21^st^ October; and 9^th^ November. The number of sampling dates was settled until the harvest of olives by olive grove owner, which occurred one week later to the last sampling date, in order to naturally preserve the field conditions to which olive fly is up against. After collection, branches were transported at refrigeration temperatures and volatile analysis was performed in the first 24 to 48 hours.

Simultaneously, fruits were collected per tree for calculation of the maturation index, as described by Hermoso et al. [17]. Briefly, samples of 100 olive fruits (20 fruits per tree) were separated in 8 levels based on epidermis and pulp color (0 to 7). Therefore, the fruit is classified as “0” if the epidermis is green; “1” for yellowish green; “2” if the epidermis shows red spots in less than half fruit; “3” if the epidermis is red or purple in more than half fruit; “4” for black epidermis and white pulp; “5” if the epidermis is black and less than half pulp is purple; “6” if the epidermis is black and more than half pulp purple (without reaching the stone); “7” if the epidermis is black and total pulp purple (reaching the stone). The maturation index was calculated as follows: MI = (*a* × 0 + *b* × 1 + *c* × 2 + *d* × 3 + *e* × 4 + *f* × 5 + *g* × 6 + *h* × 7) / 100, where the letters are the number of fruits in each level of classification considered.

To assess infestation level, from 4^th^ August to 23^rd^ November, 20 random handpicked fruits were collected fortnightly from each olive tree (5 trees per cultivar; 100 fruits) and inspected in a binocular stereomicroscope for signs of infestation (oviposition sites or exit holes). Infestation level was expressed as the percentage of infested olive fruits.

### Volatile characterization

#### SPME fibers

For the headspace solid-phase microextraction (HS-SPME) a fiber coated with divinylbenzene/carboxen/polydimethylsiloxane (DVB/CAR/PDMS; 50/30 μm) was selected based on a preliminary assay conducted with further two fibers (CAR/PDMS 75 μm and PDMS 100 μm), all from Supelco (Bellefonte, USA). Selection of the fiber was based on the highest qualitative (number of volatiles extracted) and quantitative data (peak areas) of a sample of olives from *cv*. Cobrançosa.

#### HS-SPME

The HS-SPME was carried out according to the methodology applied by our research group in other matrices [18], with some modifications. Healthy olives (one per replicate) were placed in 50 ml vials, deuterated chloroform (99.96%, Aldrich) was added as internal standard (250 ppm in methanol; 10 μl) and immediately sealed with a polypropylene cap with silicon septum. The volatiles were released at 40°C during 30 min, in an ultrasonic bath. After that, the DVB/CAR/PDMS fiber was exposed during 1 hour at 40°C for volatiles adsorption, and then inserted into the injection port of the GC system for thermal desorption and reconditioning (10 min at 280°C). For each harvest moment and olive cultivar the HS-SPME analysis was performed in quintuplicate (five different olives).

#### Gas chromatography-mass spectrometry (GC-MS) conditions

Chromatographic analysis was performed on an Agilent 6890 series GC (Agilent, Avondale, PA, USA), with splitless injection, coupled to a MS detector (Agilent 5973). Volatiles were separated using a bonded phase fused-silica capillary column (SPB-5, 60 m × 0.32 mm × 1 μm, Supelco, Bellefonte, USA), operating at constant flow with helium at 1 ml min^-1^. The oven temperature program was isothermal for 5 min at 40°C, raised to 220°C at a rate of 3°C min^-1^ and maintained at 220°C for 2 min, with a total run of 67 min. The transfer line to the mass spectrometer was maintained at 250°C. Mass spectra were obtained in electronic impact mode at 70 eV, with a multiplier voltage of 2056 V, collecting data at a rate of 1 scan s^-1^ over the range 30–500 *m/z*. The constituents were identified by comparing the experimental spectra with spectra from NIST 98 data bank (NIST/EPA/NISH Mass Spectral Library, version 1.6, U.S.A.), and also by comparison of their GC retention index [19]. Retention indices were obtained using a commercial n-alkanes series C_7_-C_30_ (Sigma-Aldrich, St. Louis, U.S.A.) by direct splitless liquid injection (1 μL) while all further conditions of GC and MS as settled for the volatile analysis. Retention indices were calculated according to van Den Dool and Kratz [20]. The compounds on Tables 1 to 3 are expressed on the basis of the relative areas achieved for the individual base ions (m/z 100% intensity). For semi-quantification purposes, total volatile amounts were calculated by the ratio of each individual base ion peak area to the area of the internal standard and converted to mass equivalents on the basis on the internal mass added.

**Table 1:** Volatile composition (relative %; mean ± standard error) of cv. Cobrançosa olives at different harvest times.

		18^th^ Jul	18^th^ Aug	20^th^ Sep	4^th^ Oct	21^st^ Oct	9^th^ Nov	
**Maturation index**		0	0	1	1	2	4	
**Chemical class**	**Compound**							***P*-value**
Alcohols								
	3-methyl-1-butanol	-	-	23.0 ± 1.3	-	-	-	-
	2-methyl-1-butanol	-	-	12.6 ± 0.7	-	-	-	-
	(*Z*)-3-hexen-1-ol	5.8 ± 1.2 a	-	9.1 ± 2.1 a	25.7 ± 5.4 b	6.2 ± 0.3 a	5.0 ± 0.8 a	< 0.001^(1)^
	Octanol	-	-	-	-	-	0.9 ± 0.1	-
Aldehydes								
	Hexanal	5.9 ± 1.1 a	11.0 ± 1.0 b	6.5 ± 0.7 a	-	4.3 ± 0.2 a	4.4 ± 0.6 a	< 0.001^(1)^
	Heptanal	-	-	-	-	2.26 ± 0.1 a	3.12 ± 0.3 b	0.036^(1)^
	Benzaldehyde	0.7 ± 0.1 a	-	-	3.5 ± 0.4 b	-	4.6 ± 0.6 b	< 0.001^(2)^
	Octanal	-	-	-	2.7 ± 0.2 a	5.0 ± 0.3 b	5.4 ± 0.8 b	0.001^(2)^
	Nonanal	-	8.6 ± 0.4 b,c	5.8 ± 0.6 a	10.3 ± 0.8 c	9.5 ± 0.7 c	6.3 ± 0.6 a,b	< 0.001^(1)^
	Decanal	-	7.5 ± 0.4 c	2.3 ± 0.2 a	4.8 ± 1.0 b	4.5 ± 0.4 a,b	2.4 ± 0.3 a	< 0.001^(1)^
Esters								
	Butanoic acid methyl ester	12.6 ± 0.8 b	13.7 ± 1.1 b	4.9 ± 0.4 a	-	-	-	< 0.001^(1)^
	Butanoic acid, 3-methyl-, methyl ester	1.1 ± 0.1	-	-	-	-	-	-
	Butanoic acid, 2-methyl-, methyl ester	3.1 ± 0.3	-	-	-	-	-	-
	(*Z*)-3-hexen-1-ol acetate	8.6 ± 1.7	-	-	-	-	-	-
Ketones								
	6-Methyl-5-hepten-2-one	-	26.0 ± 3.7 b	-	-	-	1.8 ± 0.2 a	< 0.001^(1)^
Sesquiterpenes								
	α-Copaene	1.7 ± 0.1a	4.2 ± 0.2 b	2.4 ± 0.2 a	3.3 ± 0.3 a,b	3.0 ± 0.2 a,b	6.1 ± 0.8 c	< 0.001^(2)^
	β-Caryophyllene	0.3 ± 0.0	-	-	-	-	-	-
Terpenes								
	α -Pinene	-	-	-	2.2 ± 0.3 a	1.4 ± 0.0 a	-	0.063^(1)^
	ρ-Cymene	-	-	-	-	-	1.8 ± 0.2	-
	Limonene	0.4 ± 0.0a	5.7 ± 1.1 b	7.2 ± 1.0 b,c	8.7 ± 0.9 b-d	12.1 ± 1.1 d	10.6 ± 1.5 c,d	< 0.001^(1)^
	Eucalyptol	-	-	-	-	-	1.4 ± 0.1	-
	(L)-Menthone	-	-	-	-	-	0.9 ± 0.1	-
	Menthol	-	-	-	-	1.8 ± 0.2 a	3.4 ± 0.4 b	0.010^(1)^
Aromatic hydrocarbons								
	Toluene	46.4 ± 1.7d,e	20.4 ± 2.3 a	26.3 ± 2.7 a,b	38.8 ± 2.9 c,d	49.8 ± 2.4 e	35.3 ± 1.4 b,c	< 0.001^(1)^
	*para*-Xylene	8.8 ± 1.5 b	2.9 ± 0.5 a	-	-	-	6.6 ± 0.7 a,b	0.004^(1)^
	*ortho*-Xylene	4.6 ± 0.7	-	-	-	-	-	-

In the same line, mean values with different letters differ significantly (*P* < 0.05)

^(1)^ *P* > 0.05, be means of Levene test. *P* values are those from one-way ANOVA analysis. Means were compared by Tukey’s test, since equal variances could be assumed

^(2)^ *P* < 0.05, by means of Levene test. *P* values are those from one-way Welch ANOVA analysis. Means were compared by Dunnett T3’s test, since equal variances could not be assumed

**Table 2:** Volatile composition (relative %; mean ± standard error) of cv. Madural olives at different harvest times.

		18^th^ Jul	18^th^ Aug	20^th^ Sep	4^th^ Oct	21^st^ Oct	9^th^ Nov	
**Maturation index**		0	0	1	1	2	4	
**Chemical class**	**Compound**							***P*-value**
Alcohols								
	(*Z*)-3-hexen-1-ol	5.2 ± 0.7 a,b	2.8 ± 0.4 b	14.8 ± 1.7 c	-	7.7 ± 1.2 b	1.5 ± 0.2 a	< 0.001^(2)^
	Hexanol	-	-	-	-	5.5 ± 1.1 b	1.8 ± 0.2 a	0.010^(1)^
	Octanol	-	-	-	-	-	0.5 ± 0.0	-
Aldehydes								
	Hexanal	2.9 ± 0.4 a,b	8.2 ± 1.5 c	5.2 ± 0.9 b,c	2.9 ± 0.2 a,b	1.7 ± 0.1 a	1.3 ± 0.2 a	< 0.001^(2)^
	Benzaldehyde	0.7 ± 0.1 a	1.3 ± 0.2 a	-	-	-	2.2 ± 0.3 b	0.001^(1)^
	Octanal	-	-	-	2.2 ± 0.2 a	-	1.7 ± 0.2 a	0.125^(1)^
	Nonanal	-	3.8 ± 0.2 a	3.6 ± 0.4 a	6.5 ± 0.8 b	3.4 ± 0.2 a	4.8 ± 0.4 a,b	0.001^(1)^
	Decanal	-	3.1 ± 0.1 c	1.5 ± 0.2 a,b	2.6 ± 0.2 c	1.8 ± 0.1 b	1.0 ± 0.1 a	< 0.001^(1)^
Esters								
	Butanoic acid methyl ester	1.6 ± 0.1 a	6.6 ± 0.8 b	3.4 ± 0.2 a,b	-	-	-	< 0.001^(2)^
	(*Z*)-3-hexen-1-ol acetate	69.5 ± 2.8 c	19.6 ± 3.4 a,b	28.7 ± 6.6 b	-	11.1 ± 2.2 a	-	< 0.001^(1)^
Ketones								
	6-Methyl-5-hepten-2-one	-	11.4 ± 0.6 b	-	4.8 ± 0.3 a	4.4 ± 0.1 a	-	< 0.001^(2)^
Sesquiterpenes								
	α-Cubebene	0.8 ± 0.1 a	1.4 ± 0.3 a	0.8 ± 0.1 a	-	-	-	0.319^(2)^
	(+)-Cycloisosativene	-	-	0.7 ± 0.2 a	2.6 ±c 0.2	1.3 ± 0.1 a,b	1.8 ± 0.2 b	< 0.001^(1)^
	α-Copaene	4.5 ± 0.4 a	7.9 ± 1.6 a,b	12.3 ± 2.5 b,c	28.7 ± 1.9 e	17.5 ± 1.5 c,d	22.4 ± 1.3 d,e	< 0.001^(1)^
	α-Muurolene	-	-	-	-	1.1 ± 0.1	-	-
	β-Caryophyllene	0.4 ± 0.1	-	-	-	-	-	-
	α-Farnesene	-	-	-	-	-	0.5 ± 0.0	-
	Δ-Cadinene	0.5 ± 0.1 a	1.1 ± 0.2 b	0.6 ± 0.1 a	-	-	-	0.009^(1)^
Terpenes								
	α-Pinene	-	-	-	2.6 ± 0.3 b	-	1.4 ± 0.1 a	0.010^(1)^
	ρ -Cymene	-	-	-	-	-	1.3 ± 0.1	-
	Limonene	0.6 ± 0.1 a	16.2 ± 2.1 c	6.2 ± 1.1 b	4.7 ± 0.3 a,b	1.0 ± 0.1 a	7.5 ± 0.8 b	< 0.001^(2)^
	Eucalyptol	-	-	-	-	-	1.0 ± 0.1	-
	Eucalyptol	-	-	-	-	-	3.0 ± 0.4	-
	(L)-Menthone	-	-	-	-	-	0.6 ± 0.1	-
	Menthol	-	-	-	-	-	1.9 ± 0.1	-
Aromatic hydrocarbons								
	Toluene	5.2 ± 0.9 a	15.5 ± 3.5 a,b	16.3 ± 2.3 b	42.4 ± 2.7 c	43.4 ± 2.0 c	37.6 ± 2.2 c	< 0.001^(1)^
	*para*-Xylene	5.5 ± 1.1 b	1.0 ± 0.0 a	5.9 ± 0.6 b	-	-	6.0 ± 0.4 b	< 0.001^(1)^
	*ortho*-Xylene	2.6 ± 0.4	-	-	-	-	-	-

In the same line, mean values with different letters differ significantly (*P* < 0.05)

^(1)^ *P* > 0.05, be means of Levene test. *P* values are those from one-way ANOVA analysis. Means were compared by Tukey’s test, since equal variances could be assumed

^(2)^ *P* < 0.05, by means of Levene test. *P* values are those from one-way Welch ANOVA analysis. Means were compared by Dunnett T3’s test, since equal variances could not be assumed

**Table 3:** Volatile composition (relative %; mean ± standard error) of cv. Verdeal Transmontana olives at different harvest times.

		18^th^ Jul	18^th^ Aug	20^th^ Sep	4^th^ Oct	21^st^ Oct	9^th^ Nov	
**Maturation index**		0	0	1	1	1	3	
**Chemical class**	**Compound**							***P*-value**
Alcohols								
	3-methyl-1-butanol	-	1.2 ± 0.1 a	17.2 ± 0.4 b	-	-	-	< 0.001^(2)^
	2-methyl-1-butanol	-	-	9.5 ± 0.2	-	-	-	-
	(*Z*)-3-hexen-1-ol	3.6 ± 0.5 a	3.9 ± 0.7 a	7.2 ± 1.6 a	17.2 ± 1.1 b	4.4 ± 0.3 a	5.7 ± 0.9 a	< 0.001^(1)^
	Hexanol	-	2.4 ± 0.3 a	-	-	-	4.0 ± 0.1 b	0.002^(1)^
Aldehydes								
	Hexanal	3.8 ± 0.5 a,b	19.9 ± 1.3 c	6.0 ± 1.2 b	-	1.7 ± 0.1 a	4.0 ± 0.5 a,b	< 0.001^(2)^
	Heptanal	-	5.0 ± 0.2	-	-	-	-	-
	Nonanal	-	11.8 ± 0.7 c	5.0 ± 0.6 b	3.3 ± 0.3 b	4.1 ± 0.2 b	1.4 ± 0.1 a	< 0.001^(2)^
	Decanal	1.0 ± 0.1	-	-	-	-	-	-
Esters								
	Butanoic acid methyl ester	17.3 ± 1.3b	3.3 ± 0.1 a	5.1 ± 0.3 a	3.5 ± 0.1 a	-	-	< 0.001^(2)^
	Butanoic acid, 2-methyl-, methyl ester	8.9 ± 1.6	-	-	-	-	-	-
	Hexanoic acid methyl ester	14.7 ± 2.8	-	-	-	-	-	-
	(*Z*)-3-hexen-1-ol acetate	9.0 ± 1.0 a	10.1 ± 0.6 a	-	-	-	-	0.414^(1)^
Ketones								
	6-Methyl-5-hepten-2-one	-	5.5 ± 0.1 b	-	2.6 ± 0.2 a	-	-	< 0.001^(1)^
Sesquiterpenes								
	α-Cubebene	0.8 ± 0.1	-	-	-	-	-	-
	α-Copaene	3.6 ± 0.5 a,b	2.5 ± 0.3 a	3.9 ± 0.3 a,b	3.7 ± 0.4 a,b	5.0 ± 0.3 b	10.2 ± 1.1 c	< 0.001^(2)^
	β-Caryophyllene	2.6 ± 0.3	-	-	-	-	-	-
	α-Farnesene	2.3 ± 0.2	-	-	-	-	-	-
	Δ-Cadinene	0.6 ± 0.1	-	-	-	-	-	-
Terpenes								
	α-Pinene	-	0.9 ± 0.1 a	-	1.0 ± 0.1 a	-	-	0.317^(1)^
	Limonene	1.0 ± 0.1 a	11.2 ± 0.5 d	7.6 ± 0.6 c	2.4 ± 0.1 a,b	2.0 ± 0.1 a,b	2.7 ± 0.2 b	< 0.001^(2)^
	Menthol	-	-	-	-	1.1 ± 0.1	-	-
Aromatic hydrocarbons								
	Toluene	11.1 ± 1.0 a	21.2 ± 1.7 b	38.6 ± 1.0 c	66.3 ± 1.0 d	81.7 ± 0.4 e	66.8 ± 0.5 d	< 0.001^(1)^
	*para*-Xylene	12.0 ± 1.6 c	1.3 ± 0.4 a	-	-	-	5.2 ± 0.3 b	< 0.001^(2)^
	*ortho*-Xylene	7.7 ± 0.7	-	-	-	-	-	-

In the same line, mean values with different letters differ significantly (*P* < 0.05)

^(1)^ *P* > 0.05, be means of Levene test. *P* values are those from one-way ANOVA analysis. Means were compared by Tukey’s test, since equal variances could be assumed

^(2)^ *P* < 0.05, by means of Levene test. *P* values are those from one-way Welch ANOVA analysis. Means were compared by Dunnett T3’s test, since equal variances could not be assumed

### Statistical analysis

#### Analysis of variance

An analysis of variance (ANOVA) with Type III sums of squares was performed using the GLM (General Linear Model procedure) of the SPSS software, version 21.0 (IBM Corporation, New York, U.S.A.). The fulfilment of the ANOVA requirements, namely the normal distribution of the residuals and the homogeneity of variance, were evaluated by means of the Kolmogorov-Smirnov with Lilliefors correction (if n>50) or the Shapiro-Wilk`s test (if n<50), and the Levene´s tests, respectively. All dependent variables were analysed using a one-way ANOVA with or without Welch correction, depending if the requirement of the homogeneity of variances was fulfilled or not. The main factor studied were the changes in volatile composition of olives from three different olive cultivars during crop maturation. If a statistical significant effect was found, means were compared using Tukey´s honestly significant difference multiple comparison test or Dunnett T3 test also depending if equal variances could be assumed or not. All statistical tests were performed at a 5% significance level.

#### Principal component analysis

Principal components analysis (PCA) was applied for reducing the number of variables in olives from cvs Cobrançosa, Madural and Verdeal Transmontana (variables corresponding to the amount of total volatiles, most abundant volatile compounds identified, and olives infestation levels during the olives maturation; overall 24 variables), to a smaller number of new derived variables (principal components or factors) that adequately summarize the original information, i.e., the effect of harvest period and olive cultivar on the volatile composition of olives from different olive cultivars with different susceptibility degrees to olive fly. Moreover, it allowed recognizing patterns in the data by plotting them in a multidimensional space, using the new derived variables as dimensions (factor scores). PCA was performed by using SPSS software, version 21.0 (IBM Corporation, New York, U.S.A.).

## Results

### Infestation levels

**Fig 1:**
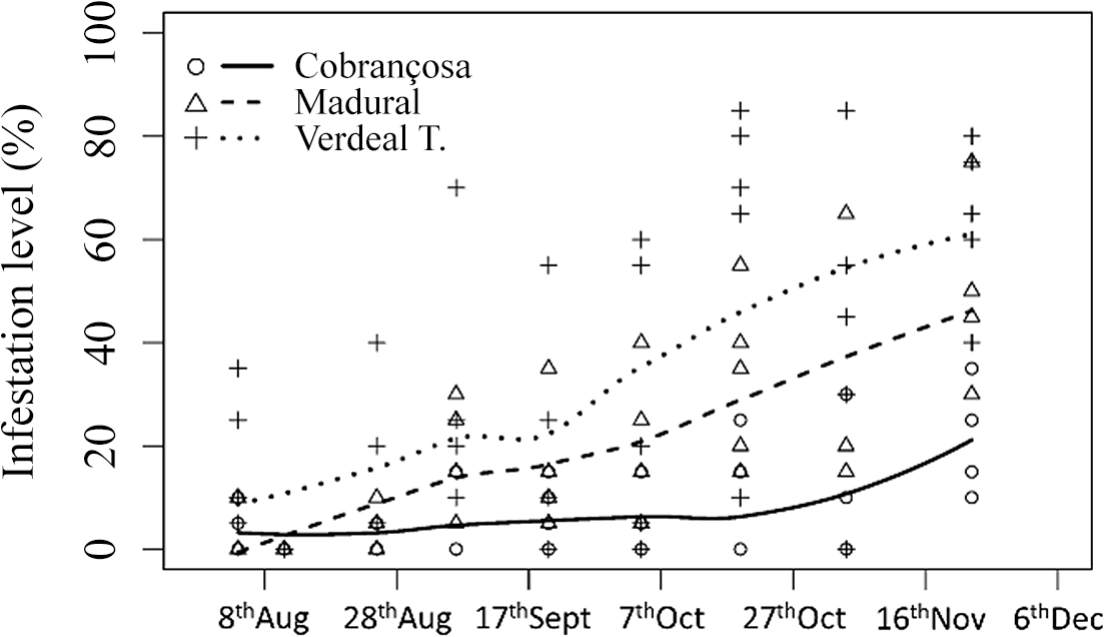
Olive fly infestation level (%) in olives from cvs. Cobrançosa, Madural and Verdeal Transmontana during crop maturation.

Infestation of olives in the three cultivars was monitored from early August until harvest, end of November). The results obtained clearly showed preference of olive fly females to lay their eggs in olives from cv. Verdeal Transmontana, followed by cv. Madural, while Cobrançosa was the less susceptible olive cultivar during the entire assessed period (Fig 1). Verdeal Transmontana displayed higher infestation levels during the entire study, reporting 16% of infestation at 24^th^ Aug, increasing continuously until mid-October, reporting 62% of olives infested. At the end of monitoring Verdeal Transmontana olives reported the highest infestation level, 64% (Fig 1). Olives from cv. Madural reported initially a low infestation of 2%, increasing steadily until 46% of infested olives at the end of monitoring. Olives from cv. Cobrançosa reported infestation levels below 10% until the 9^th^ Nov (Fig 1), while the highest infestation level was verified at the end of the assessed period, with 22% infestation levels, three times less than cv. Verdeal Transmontana, and half of infestation verified at cv. Madural.

### Volatile content and characterization

Volatile composition of olives from cvs. Cobrançosa, Madural and Verdeal Transmontana were assessed by HS-SPME-GC/MS during crop maturation at six sampling dates. The detailed relative volatile composition is reported in Tables 1–3 for cv. Cobrançosa, cv. Madural, and cv. Verdeal Transmontana, respectively. A total of 34 volatile compounds were identified in the three olive cultivars, distributed by 7 chemical classes: alcohols (5); aldehydes (6); aromatic hydrocarbons (3); esters (5); ketone (1); sesquiterpenes (7); and terpenes, more specifically monoterpenes (7).

**Fig 2:**
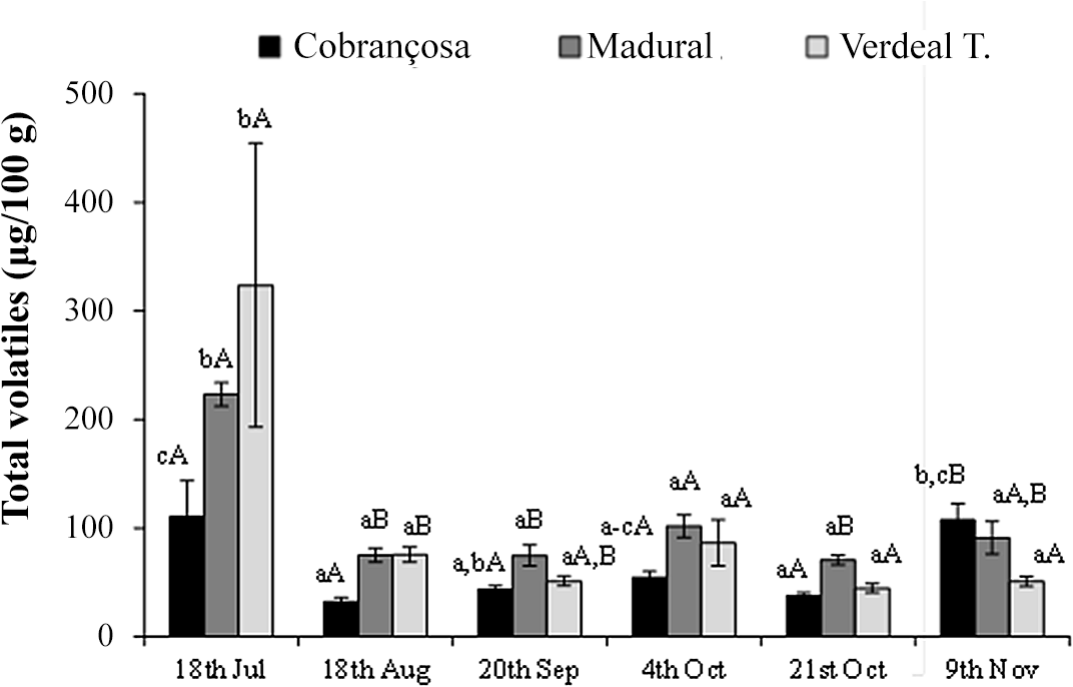
Total volatiles emission (μg 100 g^-1^ of olives) of cvs. Cobrançosa, Madural and Verdeal Transmontana olives at different harvesting times (18^th^ Jul; 18^th^ Aug; 20^th^ Sep; 4^th^ Oct; 21^st^ Oct; 9^th^ Nov) during fruit maturation (in each cultivar different minor letters represent significant differences during crop maturation (*P* < 0.05); in each harvest moment, capital letters represent significant differences between olive cultivars (*P* < 0.005)).

The semi-quantification of total volatiles revealed always higher emissions in olives from cvs. Verdeal Transmontana and Madural than cv. Cobrançosa, except for the last sampling date, 9^th^ Nov (Fig 2). At the first sampling date, Verdeal Transmontana olives emitted on average a total of approximately 324 μg of volatiles per 100 g of olives, while Madural and Cobrançosa olives reported, 223 and 111 μg per 100 g, respectively. Generally, a sharp decrease was observed in all cultivars at the second sampling date (18^th^ Aug), with a slight increase on the 4^th^ Oct., and again on the last sampling date (9^th^ Nov), similar in the three cultivars. Despite the lower volatile amounts in cv. Cobrançosa olives at all sampling dates, in the last one a higher volatile content was emmited, with 108 μg 100 g^-1^, while cv. Madural and cv. Verdeal Transmontana had only 91 and 51 μg 100 g^-1^, respectively (Fig 2). Therefore, and independently from the volatile identities that will be further explored in the next paragraphs, the total volatile amounts seem to have an attractive effect to olive fly oviposition.

**Fig 3:**
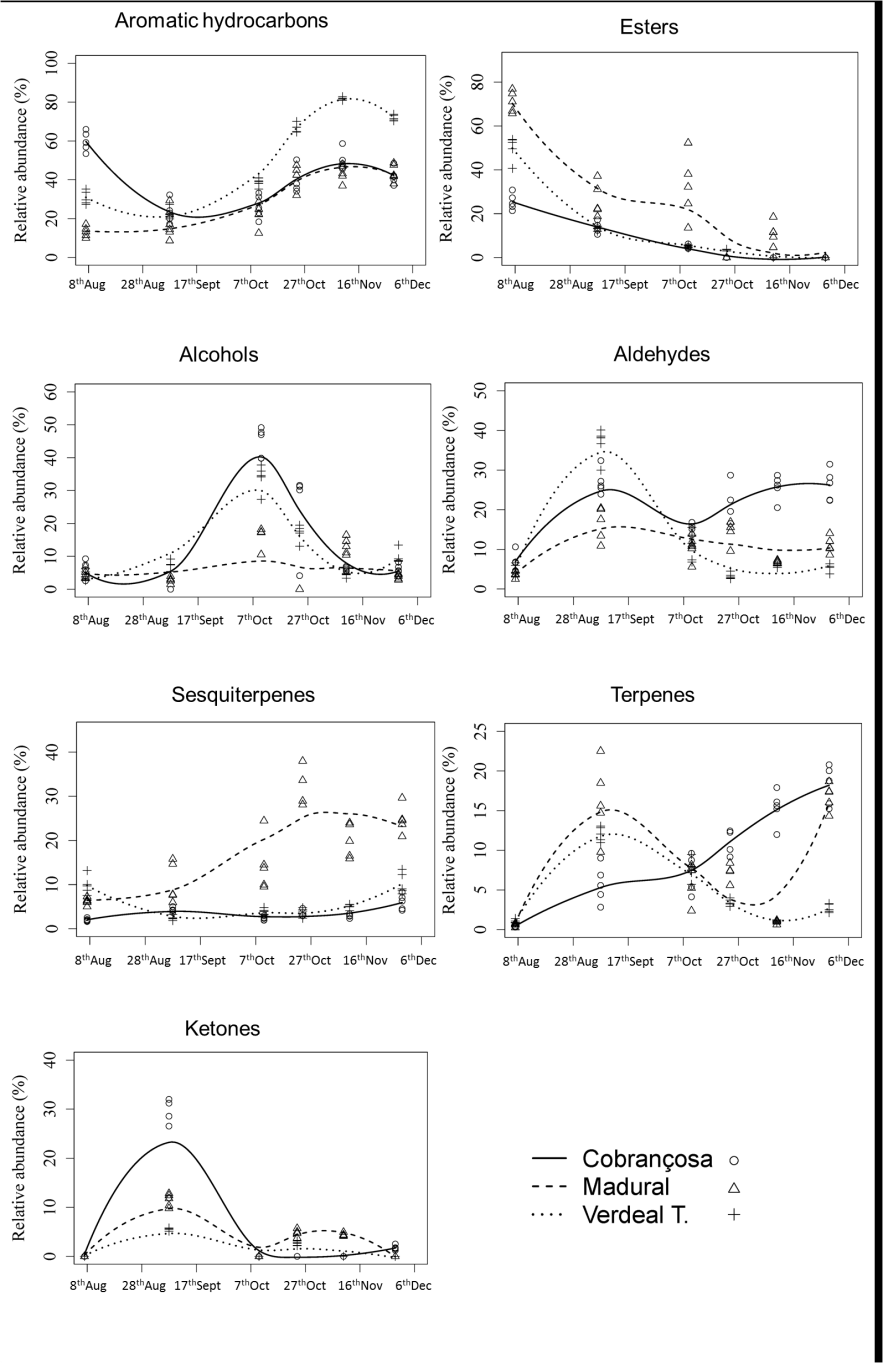
Volatile relative changes in the chemical classes identified in olives from cvs. Cobrançosa, Madural and Verdeal Transmontana during crop maturation.

Regarding volatile composition, aromatic hydrocarbons were present in the three olive cultivars (Fig 3), represented mainly by toluene, followed by *para*- and *ortho*-xylene. In the case of *ortho*-xylene, it was found only at the first sampling date (18^th^ Jul) in the three olive cultivars (Tables 2 and 3) while its isomer was only detected at the first two sampling dates (18^th^ Jul and 18^th^ Aug) in cvs. Cobrançosa and Verdeal Transmontana, being persisted to the 20^th^ Sep (third sampling date) in cv. Madural and reappearing in the last sampling date (9^th^ Nov). In opposition, toluene was present during the entire study, with significant variations in the three olive cultivars (*P* < 0.001) (Tables 1–3). The trend observed in the relative area proportion of aromatic hydrocarbons during crop maturation was similar for cvs. Madural and Verdeal Transmontana, increasing until 4^th^ Oct, and then decreasing to the 9^th^ Nov. In the case of cv. Cobrançosa it was already heavily represented in the first sampling date with slight variations over the time course studied (Fig 3).

Esters were highly represented in the first sampling dates in cvs. Cobrançosa and Verdeal Transmontana, while in the last sampling dates they were almost absent (Tables 1–3), following apparently a similar trend in the three olive cultivars with a relative decrease during ripening (Fig 3). A particular note to butanoic acid methyl ester in Cobrançosa and Verdeal transmontana, and to (*Z*)-3-hexen-1-ol acetate, particularly detected in cv. Madural (Table 3).

Alcohols trend during olives maturation was also similar in the three cultivars. Alcohols relative abundance increased form 18^th^ Jul until 20^th^ Sep with significant decreases onwards (Fig 3), being present in lower relative abundance in cv. Madural almost during the entire study (except at 21^st^ Oct). The predominant alcohols were (Z)-3-hexen-1-ol and 3-methyl-1-butanol. The first varied significantly during fruits maturation in the three cultivars (*P* < 0.001). In the case of 3-methyl-1-butanol, it was absent in cv. Madural, being present in cv. Cobrançosa only at 20^th^ Sep and in cv. Verdeal Transmontana at 18^th^ Aug and 20^th^ Sep.

Aldehydes were one of the most diversified and consistent classes of volatile compounds found in the olives analyzed. In the three cultivars studied aldehydes reported higher relative abundance at 18^th^ Aug (Fig 3). From 18^th^ Aug until the end of the study, 9^th^ Nov, aldehydes decrease their relative content in cvs. Madural and Verdeal Transmontana, while in cv. Cobrançosa aldehydes remained practically constant. Hexanal, nonanal and decanal were the most representative aldehydes present in the olives.

For sesquiterpenes a cultivar effect was observed. Significant higher values (*P* < 0.001) were reported for cv. Madural in the last three sampling dates (> 20%), with maximum relative proportions at the 4^th^ Oct (Fig 3). The most abundant sesquiterpene present in the volatile fraction of the three olive cultivars was α-copaene (Tables 1–3), being present in the three olive cultivars in all sampling periods. The highest diversity of and amounts of sesquiterpenes was detected in cv. Madural. Some of them were only present in the first sampling dates (α-cubebene; β-caryophyllene, and Δ-cadinene), while (+)-cycloisosativene was exclusive from this olive cultivar and was only detected from 20^th^ Sep onwards (Table 2). In cv. Verdeal Transmontana four sequiterpenes were identified (α-cubebene; β-caryophyllene; α-farnesene; and Δ-cadinene), however they were present only at the first sampling date (Table 3). Olives from cv. Cobrançosa had generally lower diversity and amounts (Table 1).

Similar to sesquiterpenes, terpenes presented a marked cultivar dependent trend. In cv. Cobrançosa olives their relative proportion increased continuously during olives maturation (Fig 3). In olives from cvs. Madural and Verdeal Transmontana, the terpenes increased from 18^th^ Jul to 18^th^ Aug, but decreased considerably after that. However in cv. Madural an increase is observed in the final two harvest periods, reaching similar values to those observed in the beginning of the study, an increase not attended by cv. Verdeal Transmontana olives. The trends observed are partially related to limonene content, the main terpene in the volatile fraction of the three olive cultivars. Limonene was identified during the entire study, reporting higher predominance in the first sampling periods for cvs. Madural and Verdeal Transmontana and in the last ones for cv. Cobrançosa (Tables 1–3).

Regarding ketones, only one volatile was identified, 6-methyl-5-hepten-2-one (Tables 1–3). A variable pattern was observed between cultivars and through maturation.

**Fig 4:**
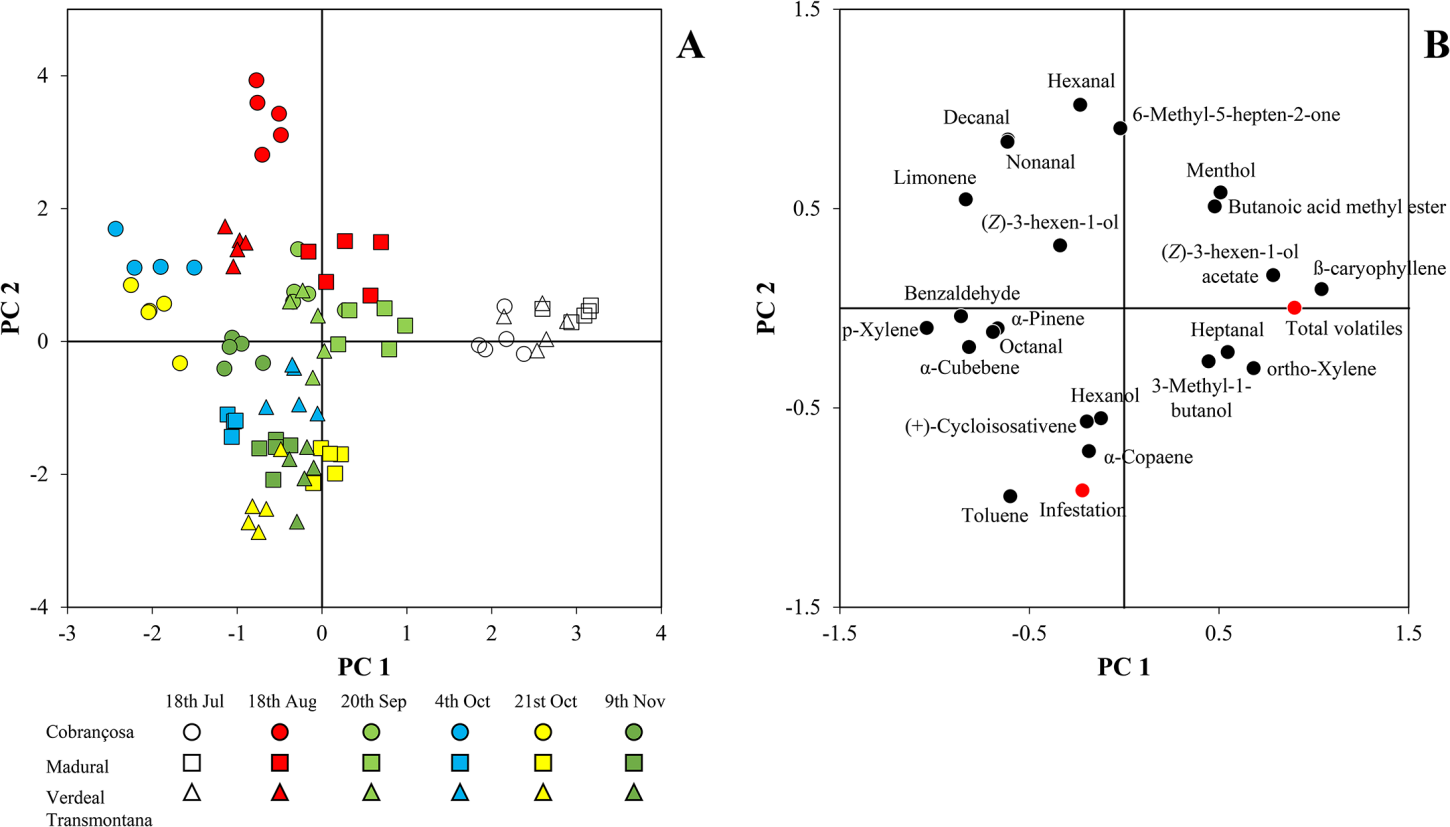
Principal component analysis obtained from the volatile composition, total volatiles and infestation levels of olives from cvs. Cobrançosa, Madural, and Verdeal Transmontana (Fig 4A) at different harvesting periods during olives maturation. The variables used in this PCA and their respective loadings are represented in Fig 4B.

In order to summarize all the information obtained, a principal component analysis (PCA) was performed with the relative proportion of the main volatile compounds identified in the olives of the three cultivars, together with the infestation levels and total volatiles emission. From the results obtained (Fig 4), the aromatic hydrocarbon toluene and the sesquiterpene α-copaene, were the two most important volatiles from olives headspace related positively with infestation levels, clearly perceptible in the PCA performed.

These two volatiles were mainly correlated with infestation level being represented in the 21^st^ Oct to 9^th^ Nov region for cvs. Madural and Verdeal Transmontana (negative regions of both principal components). These observations were are also corroborated by the results obtained in the infestation level monitored in the field, with higher infestation levels reported in the same dates and cultivars (Fig 1). If we analyze carefully the PCA obtained, samples from cv. Cobrançosa are represented in the extreme opposite region to toluene, α-copaene and infestation recorded (Fig 4). Even with the use of unsupervised statistical tools is possible to observe the notorious differentiation in the volatile composition of the three olive cultivars during olives maturation and the agglomeration of samples with higher infestation levels around toluene and α-copaene (Fig 4).

In fact these two volatiles were correlated with infestation level in the olive cultivars. In our study, the relative toluene amounts were extremely correlated with infestation level (*P* < 0.001; R^2^ = 0.256; *y* = 0.54*x* – 1.813). Only in cv. Cobrançosa toluene contents were not correlated with infestation level (*P* = 0.614; R^2^ = 0.009; *y* = -0.14*x* + 37.04). Olive cultivars Madural (*P* < 0.001; R^2^ = 0.368; *y* = 0.50*x* + 17.62) and Verdeal Transmontana (*P* = 0.003; R^2^ = 0.274; *y* = 0.49*x* + 32.55) infestation levels were extremely correlated with toluene content.

Regarding α-copaene, positive significant correlations were also established between its relative abundance in olives from cvs. Madural and Verdeal Transmontana and the infestation levels caused by olive fly during olives maturation (cv. Madural—*P* = 0.02; R^2^ = 0.168; *y* = 0.20*x* + 12.04; cv. Verdeal Transmontana—*P* = 0.05; R^2^ = 0.134; *y* = 0.04*x* + 3.688). In cv. Cobrançosa olives no correlation was observed between α-copaene emission and olives infestation level (*P* = 0.149; R^2^ = 0.073; *y* = 0.06*x* + 3.094).

In the same region where were represented α-copaene and toluene were also (+)-cycloisosativene and hexanol. The sesquiterpene was only present in cv. Madural and the alcohol was present mainly in the last sampling date in both cvs. Madural and Verdeal Transmontana. Both compounds revealed no direct correlation with infestation levels caused by olive fly.

## Discussion

The results observed in infestation levels demonstrate clearly that cv. Verdeal Transmontana is the most susceptible olive cultivar, being cv. Madural an intermediate, and cv. Cobrançosa the less susceptible to olive fly oviposition. Such differences in the susceptibility of olive cultivars could be ascribed to the volatile content and composition of the olives in conjunction with factors of other nature (physical and molecular).

Total amounts of volatiles reduced during olives maturation (Fig 2). The main contributors are GLV’s (green leaf volatiles), mainly alcohols ((*Z*)-3-hexen-1-ol), aldehydes (hexanal), and esters ((*Z*)-3-hexen-1-ol acetate). The biosynthesis of these olives components is guided by lipoxygenase pathway, also known as LOX. LOX activity starts when olive tissues are disrupted and enzymes released, taking contact with fatty acids, mainly polyunsaturated fatty acids (linoleic and linolenic). The polyunsaturated fatty acids are oxidized by LOX and cleaved by hydroperoxide lyase, forming aldehydes. The aldehydes formed are then reduced to alcohols by alcohol dehydrogenase action. Alcohols could be then esterified to yield esters due to alcohol acyltransferase. According to the polyunsaturated fatty acid intervened, different volatiles are formed. For instance hexanal is formed from linoleic acid, while (*Z*)-3-hexen-1-ol and (*Z*)-3-hexen-1-ol acetate are formed from linolenic.[21]. In our study intact olives were analyzed, therefore LOX pathway is considerably reduced due to the low/absence of tissues disruption, mainly attributed to olive fly damage [22]. This explains why in our study volatiles emission from olives is considerably low (from 32 to 324 μg per 100 g of olives). Also, the reduction in volatiles emission during olive maturation is also expected, since many of the enzymes present in LOX pathway reduce their activity during maturation, like lipoxygenase [23] and alcohol dehydrogenase [24].

Toluene is apparently one of the most abundant volatile in olives from the three cultivars, with special high relative abundance in cv. Verdeal Transmontana. This aromatic hydrocarbon, generally considered an environmental contaminant, is naturally present in the volatile profile of olive leaves and fruits [13, 25], and in olive food products: olive oil [26], and table olives of different preparation methods [27, 28]. Therefore, toluene is not a strange component in the volatile fraction of olive tree organs or olive food products. Nevertheless, the origin of toluene in olives and derived products is still controversial, since some authors believe that its presence may be due to exogenous contamination, others hypothesize the formation of this aromatic hydrocarbon by endogenous mechanisms [29], and a third part recognize the formation of aromatic hydrocarbons by the microflora naturally present in olive tree [14], with special reference to epiphytic community. Epiphytic community of olive tree is diverse [30], and according to Saccheti et al. [31] a strong relationship is observed between olive fly population and epiphytic microorganisms in olives. Therefore, olive surface epiphytic community may also be a key aspect in the olive cultivar oviposition preference of olive fly, since the survival of adults is also dependent in these communities [31].

Generally, during maturation, olives exhale higher proportions of toluene, but the amounts released are dependent on olive cultivar, being increased in the olive cultivars with higher susceptibility to olive fly (cvs. Madural and Verdeal Transmontana). For instance, in Italian olive cultivars Cellina di Nardò and Ogliarola Barese toluene amounts also increased from green to mature olives, with higher expression in cv. Ogliarola Barese [32]. The same pattern was verified by Scarpati et al. [13] that found toluene as one of the most abundant volatiles in the headspace of half-ripe olives from cv. Itrana. As we verified earlier, toluene is extremely correlated with infestation level. This observation is very interesting since olives headspace volatiles determined are recognized as being highly attractive to olive fly in attractancy bioassays [13], corroborating that some volatiles present could interfere in the behavior of olive fly. Therefore, toluene could be at least partially responsible for the behavior of olive fly. Indeed, when tested in attractancy bioassays, toluene proved a highly attractive action towards olive fly [13]. Nevertheless, toluene could act in a synergic way with others volatiles from olives.

Like toluene, the sesquiterpene α-copaene was also extremely correlated with olive fly infestation. Therefore, α-copaene presence in olives volatile fraction may as well act as an oviposition promoter for olive fly females. In fact, the role of α-copaene in the fruit susceptibility to olive fly is already recognized [16]. The amounts of α-copaene released from different cultivars and the infestation levels of olives were also correlated, in accordance to our data. Bioassays revealed that the enantiomer (+)-α-copaene is an oviposition promoter, nearly doubling the number of olives attacked by olive fly females [16]. In our study we verified that α-copaene emitted were clearly higher in cvs. Madural, followed Verdeal Transmontana, the more susceptible olive cultivars, while cv. Cobrançosa volatiles reported lower proportion of α-copaene, being this cultivar less susceptible. Therefore, based on our data and on the hypothesis that α-copaene is a potent oviposition promoter [16], this sesquiterpene could be partially responsible for the oviposition preference of olive fly towards different olive cultivars. Nevertheless, this hypothesis needs to be further exploited by field-trials and also electroantennographic studies to prove their impact in olive fly behavior.

## Conclusions

In light of the obtained results, it is clear that olive fly has an oviposition preference for cv. Verdeal Transmontana and a lower preference for cv. Cobrançosa. The volatile composition of olives is dependent on the olive cultivar, and is highly influenced by olives maturation. The different susceptibility degrees of olive cultivars could be ascribed to the volatile profile, since positive correlations were established between important components and amounts of olives headspace and the infestation recorded in each cultivar. Despite the inexistence of negative interactions, which could directly open field strategies to repulse olive fly, further studies, namely bioassays, field trials and electroantennographic studies, need to be carried out to elucidate olive fly thresholds for these volatiles and possible mechanisms of action while aiding in the development of new strategies to control olive fly populations in olive groves. Furthermore, this type of studies should be expanded to a higher number of olive cultivars in order to obtain even more reliable and strong data about the influence of volatile composition in the olive fly oviposition preference.
